# Maxillary Reconstruction with Xenogeneic Bone Graft, Platelet-Rich Fibrin, and Titanium Mesh for Rehabilitation with Implants: A 5-year Follow-Up Study

**DOI:** 10.1155/2022/3412190

**Published:** 2022-11-08

**Authors:** Graziele Parize, Samy Tunchel, Alberto Blay, Eduardo Felippe Duailibi-Neto, Yeon Jung Kim, Debora Pallos

**Affiliations:** ^1^University of Santo Amaro, São Paulo, Brazil; ^2^Private Office, São Paulo, Brazil

## Abstract

**Aim:**

Alveolar deficiency is considered one of the main limitations for placement of osseointegrated implants, as well as for their long-term success, especially in the anterior region of the maxilla.

**Objective:**

To report a clinical case of reconstruction of atrophic maxilla with deproteinized bovine bone associated with platelet-rich fibrin (PRF) and titanium mesh and to assess the linear and volumetric bone gains and rehabilitation with implants in a 5-year follow-up.

**Case:**

Patient with bone deficiency in the anterior maxilla region underwent bone reconstruction with deproteinized bovine bone associated with PRF and titanium mesh. After six months, the necessary bone height and volume were obtained for placement of implants, and the bone contour was restored in the anterior region, providing functional and aesthetic improvement. The amount of linear and volumetric bone gains was compared to baseline computed tomography scans. Three implants were placed in the grafted region, and a bone tissue sample was obtained at the time of their placement. Histological analysis showed neo-formed bone tissue in contact with the remaining particles of the biomaterial. After six months, the implants were activated, and the prosthesis was placed on the implants, which were monitored for five years.

**Conclusion:**

Implants can be placed predictably in regions with vertical and horizontal bone augmentations by using xenogeneic bone grafts associated with titanium mesh and PRF.

## 1. Introduction

Osseointegrated implants have become a great option to restore lost teeth. The available bone structure will determine the possibility of this rehabilitation [1]. Alveolar ridge resorption can restrict dental implant placement. Normally, bone resorption occurs because of tooth loss resulting from trauma, caries, and periodontal disease. Therefore, bone reconstruction procedures are performed to provide adequate conditions for rehabilitation [2].

In cases of limited amount of bone, the placement of short implants may be a solution in the posterior regions, mainly lower ones. Bone reconstruction may be the best therapeutic choice as it provides an opportunity to restore the lost bone structure, which often leads to better aesthetic results and longer implant survival [2, 3].

Autogenous block graft is considered the gold standard for these cases because it has osteoinductive, osteogenic properties. However, on the other hand, it has limited donor sites, possible morbidities, and reports of unpredictable resorption [4–8]. Therefore, more recent clinical techniques are being developed and advocated by several authors to avoid these problems [9–11].

One of the main complications of bone grafting is bone resorption. To avoid this complication, bone augmentation procedures associated with a non-resorbable membrane can be used to minimize the resorption [10].

Xenogenous bone is used for bone reconstructions with reliable results, reduced morbidity, and low complication rate compared to grafts with autogenous bone. Furthermore, they show good long-term stability due to their slow resorption, which is one of their most valuable features [12, 13].

Titanium mesh was chosen, because it has characteristics such as easy handling, allowing the three-dimensional reconstruction of large bone defects both vertically and horizontally [4, 10, 14, 15]. Platelet-rich fibrin (PRF) membrane has a slow release of growth factors, such as transforming growth factors *β*1, platelet-derived growth factor, and vascular endothelial growth factor, all allowing the improvement of wound healing and regeneration of tissues [16].

The objective of this case report was to demonstrate the feasibility of vertical and horizontal bone reconstructions through grafting with deproteinized bovine bone mineral (DBBM) associated with PRF and titanium mesh.

## 2. Case Presentation

This case report was approved by the Research Ethics Committee of the University of Santo Amaro according to protocol number CAAE 09095619000000081, and the patient read and signed the informed consent form.

**Figure 1:**
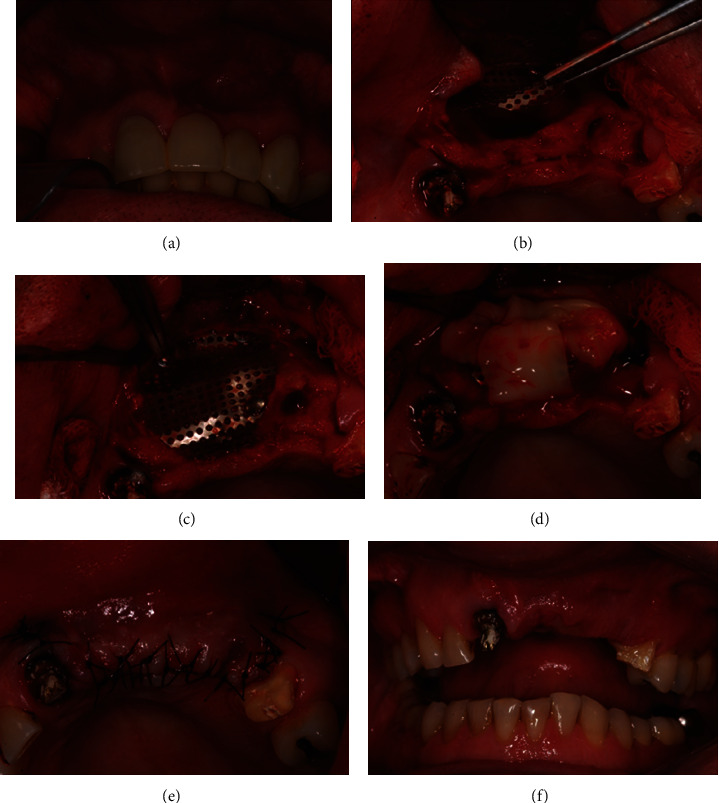
(a) Initial case. (b) Surgical bed preparation. It is possible to observe the bone defect in the region. (c) Xenogeneic bone and mesh in the region correcting the bone defect. (d) Adaptation of PRF membrane to the already-repaired tissue. (e) Surgical bed suture. (f) Removal of stitches without signs of inflammation or infection, good tissue healing.

A male patient, 56 years old, came to the dental office with the main complaint of absence of teeth #21 and #22. In the initial clinical and radiographic evaluations, it was found that tooth #23 was indicated for extraction. The initial bone and gingival appearance can be seen in Figure 1(a), which shows a depression beneath teeth #21 and #22 indicating a volumetric bone loss in this region.

Computed tomography was used to analyse the bone structure, showing that there was extensive resorption of the alveolar bone in the anterior region of the maxilla, making it impossible to place implants and requiring a reconstruction to increase bone volume and height. For analysis of the linear bone measurement, the patient's upper arch was imaged with TCX Prexion (X Trillion Inc., Tokyo, Japan). To obtain the best image quality, scans were performed at cubic field of view of 8 cm^3^, 90 kVp, 4 mA, and 37-second exposure. Next, the images were exported in Diagnostic Imaging and Communication in Medicine (DICOM) format with a voxel size of 0.160 mm. All files were individually exported to a DICOM directory to allow their visualization by using the OnDemand 3D Dental™ software (Cybermed Co., Tustin, CA, USA) for taking measurements. First, the images were captured and saved in the DICOM system for analysis of the angulation position of the patient's head (axis and reslice), followed by tracing over the region with the Arch/Curve tool to select the area of interest and obtain the measurements. A perpendicular line was also drawn along the slope of the residual bone, and three horizontal lines were recorded in width. This measurement was taken at a distance of 7, 11, and 13 mm from the crest.

The initial thickness was 2.60 mm, and the height was 16.75 mm. The patient did not present any systemic alteration contraindicating the surgical procedures.

A reconstructive surgery was performed by using Bio-Oss® bone graft (Geistlich Pharmaceutical, Wolhusen, Switzerland) as a biomaterial associated with Intralock titanium mesh (i.e., Ti Mesh-Lock® surgical mesh with 0.15 mm thick and spaced 0.8 mm holes designed for allowing cuts without edge) and PRF for obtaining the necessary amount of bone in the region for later placement of the implants and rehabilitation of the patient.

### 2.1. Site Preparation

Peripheral blood was initially collected in five vacutainer tubes, in which the white one (VACUETTE^®^ Interlock, Intra-Lock, Brasil) was centrifuged in a centrifuge (IntraSpin™, Intra-Lock Iberia, IntraSpin, Intra-Lock, FL, USA) for 3 minutes at a rotation of 2700 rpm. Next, 3 mL of the supernatant was pipetted and stored in a sterile syringe. The other four red tubes (BD Vacutainer^®^, UKMolt 2/4 Hu-Friedy - Brasil) were centrifuged for 12 minutes at 2700 rpm for preparation of PRF membranes according to the Choukroun technique [17].

The surgical procedure started by infiltrating anesthesia with articaine 4% (Ultracain D^®^, Sanofi-Aventis, Paris, France) in the region before performing a crestal incision with relaxing incisions for total detachment of the flap and full visualization of the defected region (Figure 1(b)). Next, the recipient area was prepared with small perforations by using #2 spherical drill to allow better vascularization of the bone. Once the PRF membranes were obtained, two ones were perforated and mixed with DBBM before adding 3 mL of the supernatant to this biomaterial mixture. This biomaterial was adjusted to the region by using a molt peeler (Molt 2/4 Hu-Friedy^®^), and then a 0.15 mm thick Ti mesh-Lock^®^ titanium mesh with 0.8 mm holes was placed onto the region and attached with screws measuring 1.4 and 6 mm. Next, the PRF membranes were placed over the mesh (Figures 1(c) and 1(d)). Suture with 3.0 nylon thread was performed avoiding tension in the operating region (Figure 1(e)). The patient was medicated with amoxicillin 500 mg, anti-inflammatories, dexamethasone 4 mg, and dipyrone 1 g. No changes were observed in the postoperative follow-up, and the suture was removed after 10 days (Figure 1(f)).

**Figure 2:**
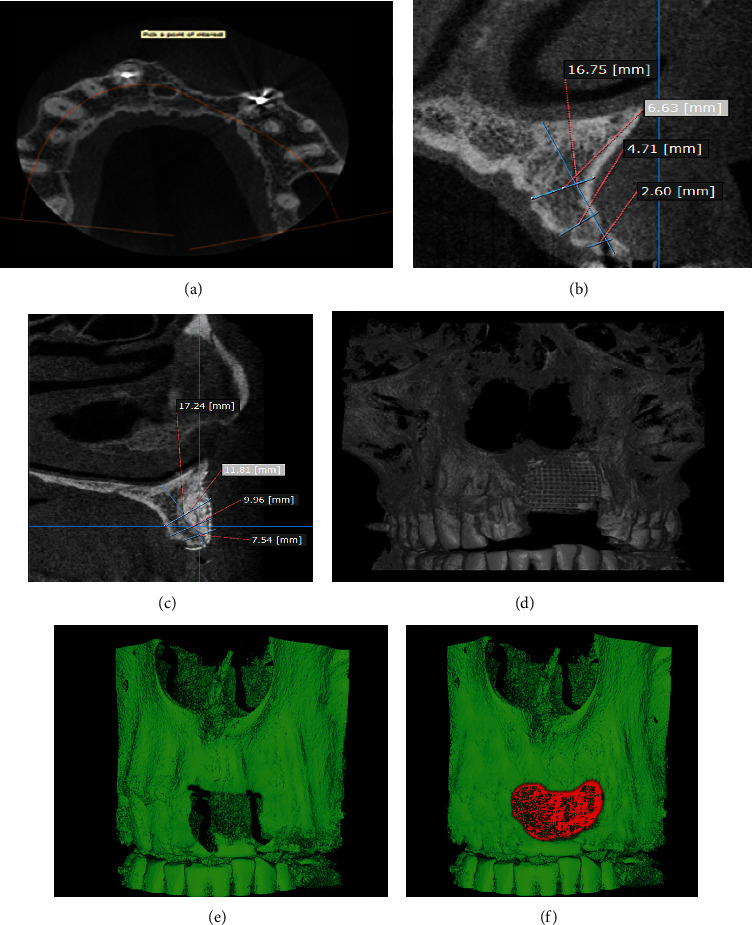
(a) Initial image showing sagittal section. (b) Initial image and linear data with measurements. (c) Final image and linear data with measurements. (d) Panoramic view with the adaptive mesh. (e) Initial volumetric image. (f) Volumetric image after grafting.

After six months, the patient underwent a new computerized tomography scan for evaluation of the bone reconstruction and implant planning (Figures 2(a), 2(b), 2(c), 2(d), 2(e) and 2(f)). The amount of bone was obtained by measuring the bone graft preoperatively and after six months approximately. Tomographic evaluation showed a thickness of 7.54 mm, height of 17.24 mm, and gain of 4.94 mm in thickness (Figures 2(b) and 2(c)).

**Figure 3:**
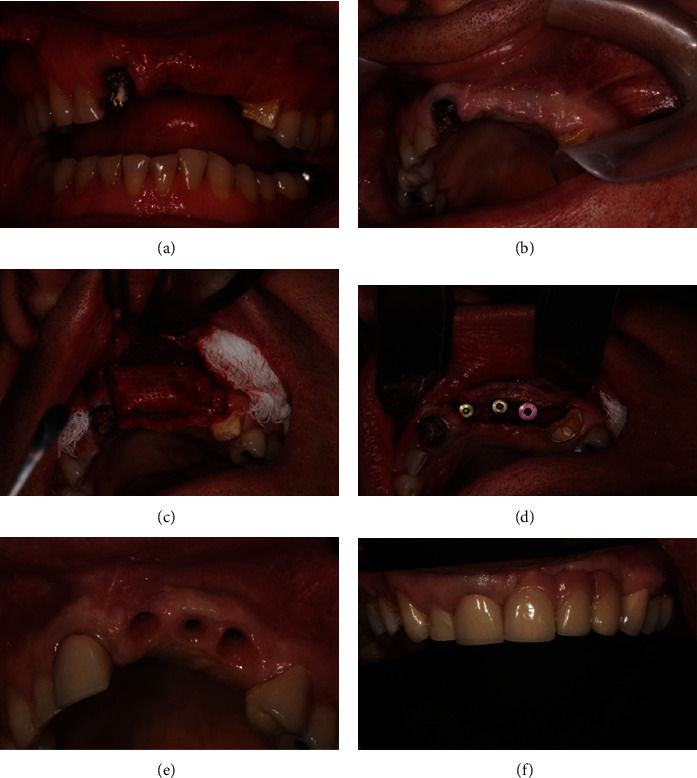
(a) Buccal visualization of the bone contour. (b) Bone and gingival contour. (c) Titanium mesh removal surgery. (d) Placement of Straumann implants. (e) Implant follow-up. (f) Permanent denture placement.

The titanium mesh was removed for performing the surgery of implant placement, and it was observed that the grafted biomaterial particles were well incorporated into the new bone (Figures 3(a), 3(b), and 3(c)). Three Straumann bone level osseointegrated implants measuring 4.1 mm × 13 mm were inserted (Figure 3(d)), after preparation of the implant placement site with a trephine drill of 2 mm in diameter and 10 in height to remove bone for histological analysis. After six months, a reopening surgery was performed by placing healing caps on each implant. Next, the patient was rehabilitated with metal-ceramic prostheses (Figures 3(e) and 3(f)).

For volumetric analysis, the patient's DICOM files obtained before and after the surgery were exported to the Slicer 5.0.3 software (2022). The images were saved as a DICOM file before being selected for allowing a multiplanar view. Four slices were selected (i.e., axial, coronal, sagittal, and volume), and the volume limit was adjusted according to the area of interest to obtain the volumetric measurement and after creating the surface. These data were obtained from DICOM files before and after the bone grafting surgery, with the difference being the volumetric gain. Therefore, it was possible in which the initial volume was 12.796 mm^3^, the volume was 54.090 mm^3^ after 6 months, and the volume was 61.913 mm^3^ on the final evaluation after 5 years (Figures 2(e) and 2(f)).

**Figure 4:**
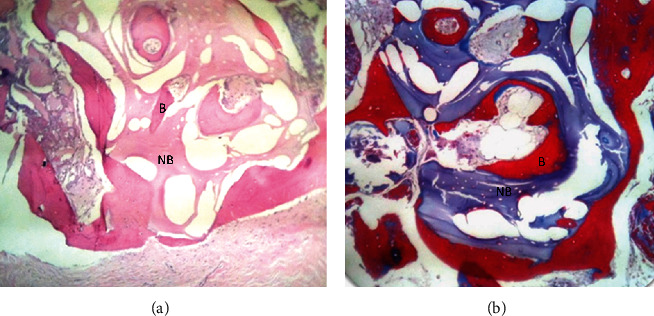
Photomicrograph showing newly formed bone in the grafted area at 6 months. (a) Hematoxylin and eosin stain (100×). (b) Masson's trichrome stain (100×). Newly formed bone was observed in intimate contact with remaining particles of biomaterial (b), and many osteocytes were distributed in the newly formed bone and, in some areas, were totally integrated, demonstrating biocompatible patterns.

**Figure 5:**
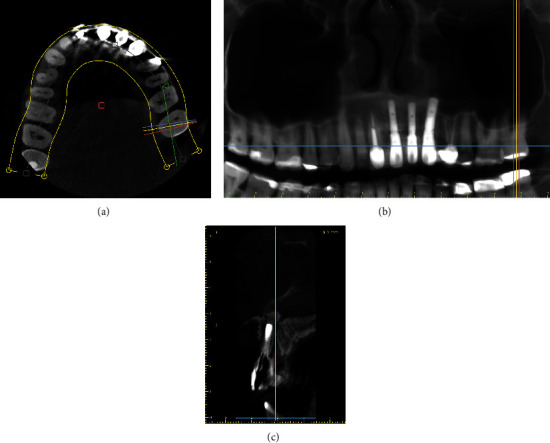
(a) Image showing sagittal section after 5 years. (b) Panoramic view showing the implant after 5 years. (c) Cone-beam computed tomography after 5-year follow-up.

At the histological analysis, a newly formed bone was observed in intimate contact with the remaining particles of the biomaterial, where many osteocytes were distributed in fully integrated areas, thus demonstrating biocompatible patterns. No inflammatory cells and multinucleated giant cells were found at the interface with new bone formation (Figures 4(a) and 4(b)).  Figure 5 shows the radiographic images of the implants installed after 5 years of follow-up.

## 3. Discussion

In this case report, it was possible to observe that bone grafting using xenogeneic bone associated with PRF membranes and placement of titanium mesh allowed rehabilitation of the patient with osseointegrated implants as height and volume were obtained. Through the analysis of preoperative and postoperative linear measurements, it was possible to observe linear gain exceeding 8 mm in some regions (Figures 2(b) and 2(c)) and volumetric gain (Figure 2(f)). The clinically obtained gingival contour culminated in a very satisfactory aesthetic result (Figures 3(a) and 3(b)). Histological analysis (Figure 4) showed that the newly formed bone was sound and well-integrated into the region, which is a good alternative compared to the autogenous bone graft.

The choice to use bone of bovine origin without association with other grafting materials was based on its advantages, such as easy availability and lower morbidity. In addition to the good results compared to other related studies, bovine bone has a lower risk of infection compared to grafting using autogenous bone [13]. Authors have suggested that the association of autogenous and xenogenous grafts at a ratio of 70 : 30 or 60 : 40 provides good results [11, 15]. In the literature, only one case report of bone reconstruction in a patient with cancer was found, in which bone grafting was performed with xenogenous bone associated with titanium mesh and PRF. The xenogeneic bone was very effective in the case of grafting of large areas, having very promising results [9, 18, 19]. According to Sanz et al. [2], xenogenous bones, also known as DBBM, are the biomaterial most used for bone grafting in the craniomaxillofacial region. The main advantages of this biomaterial lie in its architecture and geometric structure, which are similar to the human bone, and in its slow absorption capacity, which is an important clinical feature to allow the increase in bone volume [3]. As for its limitations, one can cite the lack of biological components, which restricts its activity, and the potential biological risk (e.g., disease transmission) [20, 21]. However, our study has shown that the xenogeneic bone is a good alternative for bone reconstruction with satisfactory and predictable results.

Autogenous bone is the gold standard for grafting procedures due to its characteristics of osteogenesis, osteoinduction, and osteoconduction [11]. However, this biomaterial has some disadvantages, such as high rate of resorption, collection of bone from the donor site, increased surgical morbidity, and limited amount of bone [15]. According to some studies, autogenous grafts seem to have a significantly higher resorption rate compared to xenografts, with averages of 24.4% for autogenous bone and 49% for xenogenous bone [5, 12, 13, 18, 19, 22, 23]. In the study by Proussaefs and Lozada (2006), grafts were performed with a combination of autogenous bone and Bio-Oss, and in the histological analysis, it was observed that the mixture of bone with residual particles of Bio-Oss and, in polarized microscopy, the xenograft obtained an active remodeling, and the newly formed bone appeared in firm contact along the structure, and no sign of resorption or active inflammatory process was seen [24]. The histological analysis of this case report showed that the bone particles appear to be well incorporated.

In the study by Corinaldesi et al. [20], the histological examination showed that the DBBM particles presented as granules well integrated in the regenerated bone. No sign of resorption was observed on the surface of DBBM for 8–9 months after grafting. In the literature, there is no study reporting osteoclastic activity six months after bone reconstruction.

PRF membrane was used to cover the titanium mesh in order to minimize the risk of possible exposure and contamination of the graft material, as the membrane acts on the soft tissue by accelerating the repair process [10]. According to other authors [10, 25, 26], the exposure of the titanium mesh ended up influencing the result of their study.

Boyne [27] suggested the use of titanium mesh for maxillofacial bone reconstruction, reporting positive results in the ridge augmentation obtained before the placement of dental implants.

Other authors pointed to the benefits of titanium mesh, including easy handling, biocompatibility, excellent mechanics, and rigid graft stabilization, thus allowing the treatment of all types and sizes of three-dimensional bone defects [28].

PRF membrane is obtained simply by centrifugation without anticoagulant, but containing platelets and leukocytes as well as a variety of growth factors and cytokines [29]. The key to tissue regeneration lies in its angiogenic potential, which can be explained by its three-dimensional structure, immune system control, cell recruitment potential, and ability to ensure wound closure [30].

The present study corroborates the literature by demonstrating that the bone gain obtained is predictable with less complications, allowing implant stability. This finding was also reported by Briguglio et al. [4], who performed a retrospective study on the association of xenogenous and autogenous grafts with PRF for reconstruction of vertical and horizontal bone defects in the maxilla and mandible.

The gains obtained in the above-mentioned reconstruction made it possible to rehabilitate the patient with implants and ceramic prostheses. It is possible to observe in the clinical images that the prostheses have a gingival margin, which was necessary because the patient's defect was very large and it would not be possible to rehabilitate it without doing so. This allowed the final result to be more aesthetic. In addition, it was possible to minimize the bone defect and prepare the region to receive the osseointegrated implants safely.

As far as we know, this is the first case report on the use of xenogeneic bone in association with titanium mesh and PRF for rehabilitation of patients with insufficient bone volume, presenting low morbidity, low risk of infection, and satisfactory results as well.

## 4. Conclusion

The present clinical case showed that implants can be placed in bone sites, which were vertically augmented by using only xenogenous grafts associated with titanium mesh and PRF. The result obtained was aesthetically and functionally satisfactory as the patient's oral and emotional health was improved with the use of osseointegrated implants. In addition, further follow-up studies are needed to assess whether osseointegration in this type of augmented bone can be maintained in the long term.

## Data Availability

Data supporting this research article are available from the corresponding author or first author on reasonable request.
